# SERPINB2 Is a Novel Indicator of Cancer Stem Cell Tumorigenicity in Multiple Cancer Types

**DOI:** 10.3390/cancers11040499

**Published:** 2019-04-08

**Authors:** Na-Hee Lee, Se-Ra Park, Jin Woo Lee, Soyi Lim, Seung-Ho Lee, Seungyoon Nam, Dong Young Kim, Seung Yeon Hah, In-Sun Hong, Hwa-Yong Lee

**Affiliations:** 1 Department of Health Sciences and Technology, GAIHST, Gachon University, Incheon 21999, Korea; lnahee89@gmail.com (N.-H.L.); sera0421@gachon.ac.kr (S.-R.P.); 2 Department of Molecular Medicine, School of Medicine, Gachon University, Incheon 406-840, Korea; jwlee@gachon.ac.kr; 3 Department of Health Sciences and Technology, SAIHST, Sungkyunkwan University, Seoul 06351, Korea; 4 Department of Obstetrics and Gynecology, Gachon University Gil Medical Center, Incheon 21565, Korea; soyilim@gmail.com (S.L.); miracle627@gilhospital.com (S.-H.L.); 5 Department of Genome Medicine and Science, College of Medicine, Gachon University, Incheon 21565, Korea; nams@gachon.ac.kr; 6 Department of Life Sciences, Gachon University, Seongnam 21936, Korea; 7 Department of Otolaryngology-Head and Neck Surgery, Gil Medical Center, Gachon University School of Medicine, Incheon 21565, Korea; hndyk94@gachon.ac.kr; 8 Department of Pathology, Gachon University Gil Medical Center, Incheon 21565, Korea; cjkha@gachon.ac.kr; 9 Department of Biomedical Science, Jungwon University, 85 Goesan-eup, Munmu-ro, Goesan-gun, Chungcheongbuk-do 367-700, Korea

**Keywords:** cancer stem cells, SERPINB2, tumorigenicity, stem cell-like properties

## Abstract

Drug resistance is one of the major characteristics of cancer stem cells (CSCs) and a mechanism of tumor recurrence. Therefore, selectively targeting CSCs may be an effective therapeutic strategy to overcome cancer recurrence. In the present study, we found that exposure to tumorigenic compounds significantly increased the growth potential and stem-cell-like properties of various CSCs. Early-response genes involved in tumorigenesis can be used as specific markers to predict potential tumorigenicity. Importantly, for the first time we identified, a labile tumorigenic response gene—SERPINB2—and showed that tumorigenic compound exposure more profoundly affected its expression in CSCs than in non-stem cancer cells, although both cells exhibit basal expression of SERPINB2 in multiple cancer types. Our data also revealed a strong relationship between the significantly enhanced expression of SERPINB2 and metastatic progression in multiple cancer types. To the best of our knowledge, this is the first study to focus on the functions of SERPINB2 in the tumorigenicity of various CSCs and these findings will facilitate the development of promising tumorigenicity test platforms.

## 1. Introduction

Recently, it has been proposed that a small population of cancer cells—cancer stem cells (CSCs) or cancer-initiating cell—within a tumor are one of the critical factors for drug resistance and tumor recurrence [1,2,3]. These studies suggest that more effective cancer treatment approaches need to specifically target and eliminate CSCs rather than simply reduce the bulk of the tumor. Distinct CSC populations have recently been identified in nearly all human cancers, including leukemia [4], breast cancer [5], colon cancer [6] and liver cancer [7]. Interestingly, due to their varying states of tumorigenicity, CSCs and non-stem cancer cells respond differently to the same carcinogen exposure; thus, differential tumorigenic effects might be expected. Indeed, compared to non-stem cancer cells, CSCs responded differently to the same stimuli with distinct gene expression patterns [8,9]. In this context, CSC-based screening platforms can provide valuable tumorigenic information of chemicals because their tumorigenicity is not normally detected by other non-stem cancer cell-based screening platforms.

Importantly, early changes in the gene expression profile mediated by carcinogen exposure are more likely to indicate the initiation of tumorigenic processes than are late-stage events, thus providing more sensitive and accurate information for early tumorigenic events [10]. Tumorigenic compound exposure may increase the expression levels of CSC markers and CSC sphere formation, which is highly consistent with stem cell-like phenotypes. Therefore, early-response genes involved in tumorigenic characteristics, such as clonogenic capacities and stemness-related properties, can be used as specific markers to predict tumorigenicity. In this study, to identify the early-response genes associated with possible tumorigenic processes, we compared the gene expression profiles of CSCs treated with the well-known carcinogen tetrachlorodibenzo-p-dioxin (TCDD) to those of non-treated cells. Among the genes that were analyzed, we observed a significant positive correlation between tumorigenic compound exposure and enhanced SERPINB2 levels. SERPINB2, also known as plasminogen activator inhibitor type 2 (PAI-2), has been shown to be directly related to tumor promotion and poor prognosis in various cancers such as bladder [11], colorectal [12], endometrial [13] and ovarian [14] cancers. Consistent with these findings, this gene has been associated with the development of some cancers caused by exposure to carcinogens [15,16]. Additionally, SERPINB2 has been identified as a significantly dysregulated gene that regulates the in vivo growth and survival of leukemia stem cells [17], suggesting the possibility that SERPINB2 might act as a regulator or biomarker for predicting metastatic progression in other cancer types, including breast, colorectal and liver cancers. In this study, we found that tumorigenic compound exposure profoundly enhanced the self-renewal and stem cell-like properties of multiple CSC types. We demonstrate here, for the first time, that SERPINB2 expression is significantly increased in response to various tumorigenic agents in multiple CSC types. More strikingly, we also found that SERPINB2 expression was substantially increased in multiple cancer types compared to non-tumor tissues, suggesting that SERPINB2 is a reliable marker for predicting tumorigenicity. Currently, the number of hazardous chemicals in existence is growing due to rapid industrialization, indicating the need for a better screening platform for predicting chemical tumorigenicity. Our CSC-based screening platforms can provide valuable information on newly developed chemicals by utilizing the tumorigenic response gene SERPINB2.

## 2. Results

### 2.1. TCDD Promotes the Self-Renewal and Stem Cell-Like Properties of Multiple CSC Types

**Figure 1:**
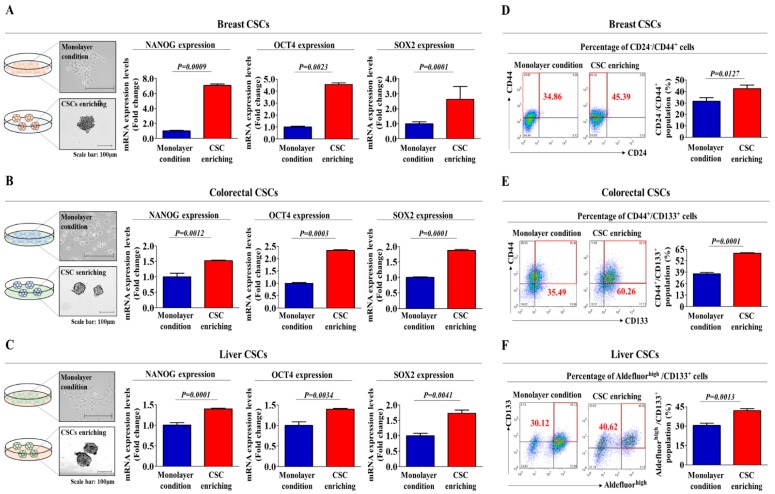
Sphere formation of cancer cells leads to the expression of stemness-related genes and cancer stem cell (CSC) markers in multiple cancer types. A 3D sphere-forming culture system was established as an in vitro culture model of multiple CSC types. Real-time PCR results demonstrated changes in the expression of the stemness-related genes NANOG, OCT4 and SOX2 after one week of sphere culture relative to the expression of human breast (MDA-MB-231), colorectal (HT29) and liver (Huh7) cancer cells cultured in subconfluent monolayers (**A**–**C**). The results of flow cytometry analysis showing the percentage of the total cell population positive for each type of CSC marker for each cancer type (Aldefluor^high^/CD133^+^ for liver CSCs; CD44^+^/CD133^+^ for colorectal CSCs; CD24^−^/CD44^+^ for breast CSCs) in both monolayer and 3D sphere cultures (**D**–**F**). The results are presented as the mean ± SD from three independent experiments.

**Figure 2:**
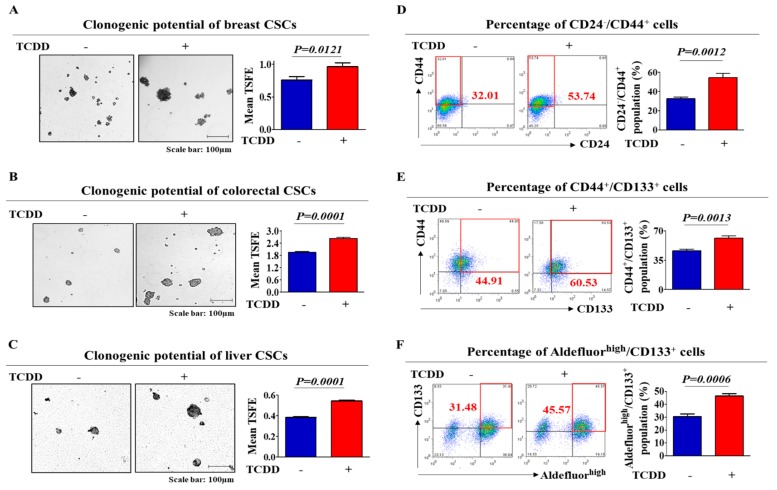
The stimulatory effects of tetrachlorodibenzo-p-dioxin (TCDD) on the self-renewal and stem cell-like properties of multiple CSC types. HT29, MDA-MB-231 and Huh7 cells were suspended in serum-free medium containing B27, EGF, bFGF and heparin and then plated onto six-well ultralow attachment dishes (1 × 10^4^ cells/well). After 48 h, the cells were treated with TCDD (10 nM) for 1 week. The stimulatory effects of TCDD on the self-renewal ability of various CSC types were assessed in sphere-forming cultures (**A**–**C**). The percentage of cells in each cancer type that were positive for the respective CSC marker and cultured with or without TCDD treatment (10 nM) was evaluated by flow cytometry analysis (**D**–**F**). Abbreviations: TSFE, Tumor sphere-forming efficiency. The results represent the mean ± SD from three independent experiments.

To determine whether tumorigenic compound exposure promotes the growth and stem-cell-like properties of multiple CSC types, we established a three-dimensional (3D)-sphere-forming culture system as an in vitro culture model of various CSC types. Previous studies have demonstrated that stem cell-like properties are enriched in non-adhesive sphere culture conditions in various cancer types, including breast [18], colon [6] and liver [19] cancers. Therefore, to confirm whether stem cell-like features are enriched in our 3D CSC culture conditions, we examined the expression patterns of the well-known stem cell markers NANOG, OCT4 and SOX2. As expected, the expression levels of these markers were higher in 3D sphere-forming cells than in monolayer-cultured cells (Figure 1A–C). To further confirm whether the non-adhesive spheroid culture system could be used to analyze the growth properties and behaviors of multiple CSCs, we performed flow cytometry analysis to quantitate the percentage of cells expressing a combination of various CSC markers for each CSC type (Aldefluor^high^/CD133^+^ for liver CSCs; CD44^+^/CD133^+^ for colorectal CSCs; CD24^−^/CD44^+^ for breast CSCs) within a total cell population. As expected, the percentage of cells expressing each CSC marker was significantly increased in the non-adhesive spheroid culture system (Figure 1D–F) compared to that in the adherent cultures. Standard tumorigenic compounds were chosen from a list of top-ranked compounds according to common hazardous material classification from five authorized international organizations: the ACGIH (Association Advancing Occupational and Environmental Health), EU (European Union), IARC (International Agency for Research on Cancer), NTP (National Toxicology Program) and US EPA (Environmental Protection Agency). The well-known carcinogen tetrachlorodibenzo-p-dioxin (TCDD) was chosen as a standard test compound because of its extremely high tumorigenicity. A schematic summary of the test compound selection is described in Figure S1. The approximate IC_50_ value of TCDD was determined by testing the responses of normal human cells to multiple concentrations of TCDD (1 µM to 100 µM) using an MTT assay (Figure S2). Next, to determine whether tumorigenic compound exposure promotes the tumorigenic potential of multiple CSC types, we evaluated the effect of TCDD treatment on CSC sphere formation and the percentage of cells that were positive for each CSC marker. The sphere-forming efficiency was significantly enhanced by TCDD treatment in various types of CSCs (Figure 2A–C). Additionally, a serial dilution assay was performed to further determine the frequency of sphere formation of multiple cancer cells following treatment with TCDD. Cancer cells treated with TCDD were seeded at 1, 10, 100 and 1000 cells/well. After 21 days of culture, the number of wells that were positive or negative for the presence of tumor spheres was determined. Consistent with earlier results, the size of the CSC spheres was significantly larger in cultures treated with TCDD, regardless of the initial cell number in the well (Figure S3A–C). Furthermore, the relative percentages of cells expressing each CSC marker were significantly increased in the TCDD-treated CSCs in multiple cancer types (Figure 2D–F) compared to those in the untreated CSCs. These results suggest that tumorigenic compound exposure profoundly stimulates the clonogenic abilities and stem cell-like properties of multiple CSC types.

### 2.2. Aberrant Activation of SERPINB2 in Response to TCDD Exposure in Multiple CSC Types

**Figure 3:**
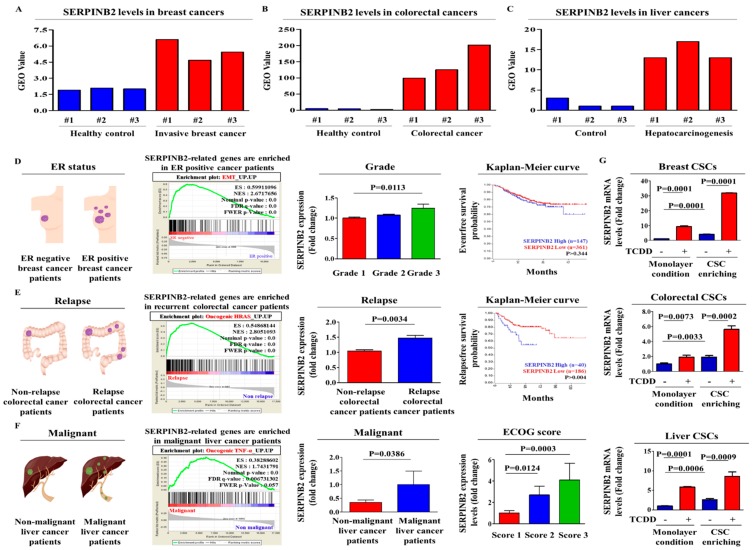
TCDD stimulates SERPINB2 expression in multiple types of CSCs and promotes its aberrant activation in metastatic progression. The Gene Expression Omnibus (GEO) database (https://www.ncbi.nlm.nih.gov/geo/) was analyzed to further verify the association of increased SERPINB2 expression with the initiation and progression of breast (**A**), colorectal (**B**) and liver (**C**) cancers. Differentially expressed genes from metastatic and non-metastatic cancers (Seiber cohort, GSE25066 for breast cancer; GSE75316 for colorectal cancer; LIHC for liver cancer) were applied to a gene set enrichment analysis (GSEA). GSEA revealed the highly enhanced expression of the SERPINB2 signaling-related gene KRAS (based on FDR *q*-value < 0.05) in breast (**D**), colorectal (**E**) and liver (**F**) cancers with metastatic progression or recurrence. The stimulatory effect of TCDD on SERPINB2 expression was assessed in both 2D adherent and 3D nonadherent spheroid cultures of multiple cancer types through real-time PCR (**G**). Abbreviations: TSFE, Tumor sphere-forming efficiency. The results represent the mean ± SD from three independent experiments.

**Figure 4:**
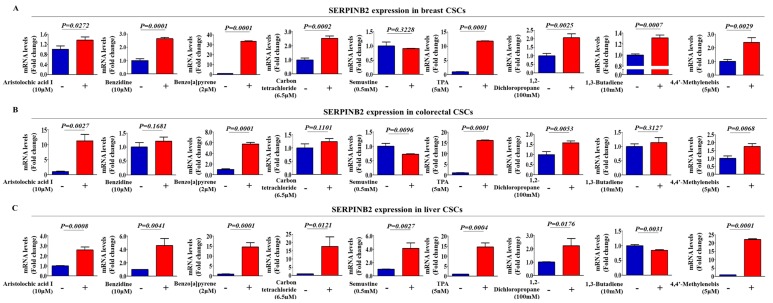
Effects of multiple test substances on SERPINB2 expression levels in multiple types of CSCs. Real-time PCR results demonstrating changes in the expression of SERPINB2 in various CSC types, including breast (**A**), colorectal (**B**) and liver cancers (**C**) with or without the treatment of multiple tumorigenic substances, including aristolochic acid I (10 µM), benzidine (10 µM), benzo[a]pyrene (2 µM), semustine (0.5 mM), TPA (5 nM), 1,3-butadiene (10 mM), 1,2-dichloropropane (100 mM) and 4,4-methylenebis (5 µM). The results represent the mean ± SD from three independent experiments.

In a previous study, we compared the gene expression profiles in TCDD-treated cells and non-treated cells to identify potential genes that were responsive to tumorigenic compound exposure using a large-scale gene expression analysis method: RNA sequencing. We observed a significant positive correlation between TCDD exposure and enhanced SERPINB2 expression among the analyzed genes [20]. The Gene Expression Omnibus (GEO) database was analyzed to verify the increased SERPINB2 expression with the initiation and progression of multiple cancer types. The expression levels of SERPINB2 were markedly enhanced with the initiation and progression of breast (Figure 3A), colorectal (Figure 3B) and liver (Figure 3C) cancers. To further clarify whether SERPINB2 expression is significantly activated in patients with metastatic progression or recurrence, we performed a gene set enrichment analysis (GSEA), an algorithm for determining whether the differentially expressed genes are enriched for particular physiological conditions, on clinical patient data from multiple cancers with the Seiber dataset (GSE25066 for breast cancer (Grade 1: 180 patients, Grade 2: 259 patients and Grade 3: 15 patients); GSE75316 for colorectal cancer (non-relapse: 99 patients and relapse: 226 patients); and LIHC for liver cancer (nonmalignant: 93 patients and malignant: 16 patients)). GSEA revealed a strong relationship between the significantly enhanced expression of SERPINB2 and metastatic progression or recurrence in multiple cancer types, including breast (Figure 3D), colorectal (Figure 3E) and liver cancers (Figure 3F). Importantly, we also found that TCDD exposure more profoundly affected the expression of SERPINB2 in 3D-cultured CSCs than in 2D monolayer-cultured cells of multiple cancer types (Figure 3G). These results suggest that tumorigenic compound exposure more profoundly stimulates SERPINB2 expression in CSCs than in non-stem cancer cells in multiple cancer types. To further determine whether SERPINB2 can serve as a “universal” marker for predicting tumorigenic responses to any type of tumorigenic materials rather than as a response gene specific to TCDD, we investigated SERPINB2 expression profiles in various CSC types with multiple known tumorigenic substances. The approximate IC_50_ values of multiple test substances were determined using a dose-response curve (Figure S4). Importantly, enhanced SERPINB2 expression levels were detected with most of the tested tumorigenic substances (Figure 4A–C), suggesting that SERPINB2 can be used as a reliable universal marker for predicting toxicity.

### 2.3. SERPINB2 Depletion Suppresses the Stem Cell-Like Properties of Various Types of CSCs

**Figure 5:**
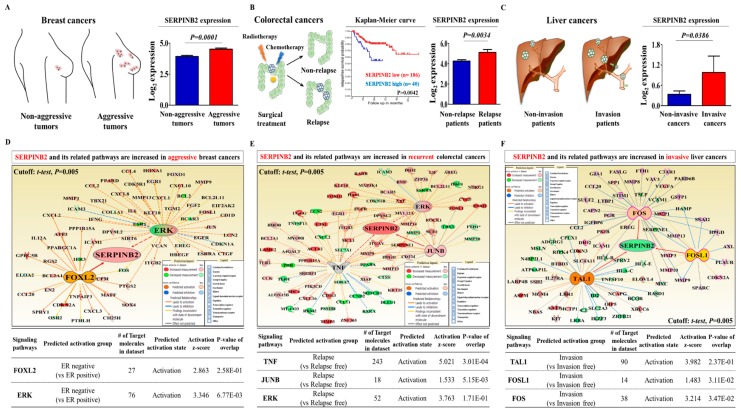
Aberrant SERPINB2 expression is associated with recurrence and metastasis of multiple cancer types. A significant correlation between tumor reoccurrence and the expression of SERPINB2 was observed in available Oncomine dataset repositories (www.oncomine.org) of breast, colorectal and liver cancers (**A**–**C**). An upstream regulator analysis was performed using Ingenuity Pathway Analysis (IPA) software (http://www.ingenuity.com) to predict the activation state (either activated or inhibited) of SERPINB2 itself and constituents of its related signaling pathways in metastatic versus non-metastatic cancers or recurrent versus nonrecurrent cases of multiple cancer types (**D**–**F**).

**Figure 6:**
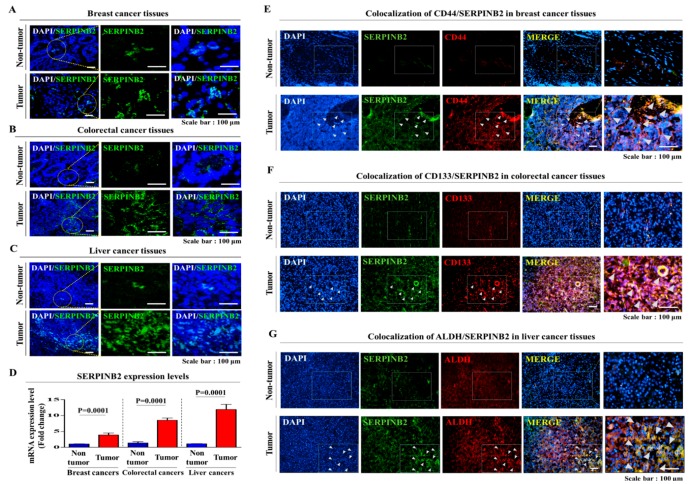
Expression profiles of SERPINB2 in human non-tumor and tumor tissues. Tumor and non-tumor tissues were stained with antibodies specific for SERPINB2 in breast, colorectal and liver cancer samples (**A**–**D**). DAPI staining was used to label the nuclei within each field. Multiple types of cancer tissues and the corresponding normal tissues were costained with antibodies specific for SERPINB2 and the CSC markers CD44, CD133 or ALDH. SERPINB2-positive cells largely overlapped with CD44-, CD133- or ALDH-positive cells in the cancerous breast, colorectal and liver tissues (**E**–**G**). The results represent the mean ± SD from three independent experiments.

**Figure 7:**
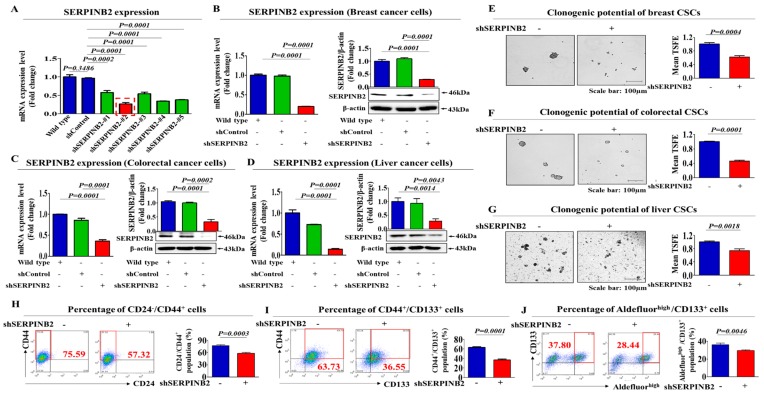
The effects of SERPINB2 knockdown on the self-renewal and stem cell-like properties of multiple CSC types. Breast cancer cells were stably transduced with shRNA #1, #2, #3, #4 or #5, all of which targeted SERPINB2 or with a nontargeting control shRNA. SERPINB2 shRNA construct #2, hereafter referred to as SERPINB2 shRNA, showed the most efficient knockdown (**A**). Successful knockdown of SERPINB2 was verified based on the RNA and protein levels in breast (**B**), colorectal (**C**) and liver (**D**) cancer cells. The results represent the mean ± SD from three independent experiments. The inhibitory effects of SERPINB2 knockdown on the self-renewal ability of various CSC types were assessed in sphere-forming cultures (**E**–**G**). The results of flow cytometry analysis showing the percentage of the total cell population that was positive for the CSC marker for the respective type of cancer (Aldefluor^high^/CD133^+^ for liver CSCs; CD44^+^/CD133^+^ for colorectal CSCs; CD24^−^/CD44^+^ for breast CSCs) with or without SERPINB2 knockdown (**H**–**J**). Abbreviations: TSFE, Tumor sphere-forming efficiency. β-Actin was used as the internal control. The results represent the mean ± SD from three independent experiments.

To investigate the relationship between poor prognosis and increased SERPINB2 levels, we analyzed the available clinical datasets for various cancer types using the Oncomine data repository. After specifically filtering the datasets for the frequency of tumor metastasis, survival or recurrence, we observed significant positive correlations between enhanced SERPINB2 levels and higher overall metastasis/recurrence rates as well as a lower 5-year survival in breast (nonaggressive: 297 patients and aggressive: 205 patients), colorectal (non-relapse: 176 patients and relapse: 50 patients) and liver (noninvasive: 93 patients and invasive: 16 patients) cancers (Figure 5A–C). Furthermore, we also analyzed the activation state of various signaling pathways and genes using Ingenuity Pathway Analysis (IPA) to evaluate the increased SERPINB2 expression itself and its related signaling regulators in metastatic versus non-metastatic cancers or recurrent versus nonrecurrent cases of multiple cancer types. In aggressive breast cancers, the expression levels of positive regulators of SERPINB2, such as FOXL2 (Z-score = 2.863, *p*-value = 0.258) and ERK (Z-score = 3.346, *p*-value = 0.677), were activated (Figure 5D). In recurrent colorectal cancers, the levels of positive regulators of SERPINB2, such as TNF (Z-score = 5.021, *p*-value = 0.301), JUNB (Z-score = 1.533, *p*-value = 0.00515) and ERK (Z-score = 3.763, *p*-value = 0.171), were also increased (Figure 5E). Finally, in invasive liver cancers, the levels of positive regulators of SERPINB2, such as TAL1 (Z-score = 3.982, *p*-value = 0.237), FOSL1 (Z-score = 1.483, *p*-value = 0.0311) and FOS (Z-score = 3.214, *p*-value = 0.0347), were increased (Figure 5F). These results suggested a strong relationship between significantly increased expression of SERPINB2 itself and constituents of its related signaling pathways and metastatic progression or recurrence in breast, colorectal and liver cancers. Consistent with the IPA results, our immunocytochemical analysis showed that SERPINB2 expression was markedly increased in tumor tissues compared to non-tumor tissues in breast, colorectal and liver cancer patients (Figure 6A–D). Additionally, to investigate the correlations between SERPINB2 and CSC populations in various cancer patients, we analyzed the co-expression of SERPINB2 in CD44-, CD133- or ALDH-positive tissue samples from breast, colorectal and liver cancer patients, respectively. As shown in Figure 6E–G, cells positive for SERPINB2 were also largely positive for CD44, CD133 or ALDH in each cancer tissue sample. Furthermore, to confirm whether SERPINB2 can mediate the tumorigenic potential of various types of CSCs, we knocked down SERPINB2 expression using a specific shRNA in breast, colorectal and liver cancer cells (Figure 7A–D). CSC sphere formation (Figure 7E–G) and the percentage of the total cell population positive for each type of CSC marker (Figure 7H,I) were significantly decreased by SERPINB2 knockdown in multiple CSC types. Additionally, a serial dilution assay was performed to further determine whether SERPINB2 knockdown can suppress sphere formation over a range of cell densities. Multiple types of cancer cells transfected with shRNA targeting SERPINB2 were seeded at 1, 10, 100 and 1000 cells/well. After 21 days of culture, the number of wells that were positive or negative for growing tumor spheres was determined. Consistently, the size of the CSC spheres was significantly suppressed by SERPINB2 knockdown in all cell densities (Figure S5A–C). These results suggest that SERPINB2 positively regulates the clonogenic abilities and stem cell-like properties of multiple CSC types.

## 3. Discussion

The current techniques for evaluating drug tumorigenicity and effectiveness largely rely on non-human, animal-based test platforms. These screening platforms offer a whole-body physiological system for predicting the effectiveness or potential tumorigenicity of chemicals or drugs. However, even advanced animal-based platforms do not faithfully mimic the physiological conditions of humans [21]. Furthermore, animal models are time consuming, ethically controversial and expensive and the results vary widely between species compared to those of in vitro models. Human cell-based in vitro screening platforms therefore offer an alternative approach to determine potential safety and efficacy [22,23,24]. Monolayer-cultured cancer cells obtained from human tissues have been the most widely used in vitro models for assessing drug effectiveness or toxicity. While these monolayer platforms have the advantages of convenience, rapidity and scalability, they sometimes provide nonpredictive tumorigenic activity and misleading data for in vivo responses [25]. Recently, multiple studies have shown that cells cultured in a 3D system provide a more physiologically relevant environment and better intercellular interactions than 2D cultures [26,27]. To determine whether tumorigenic compound exposure promotes the growth potential and stem-cell-like properties of multiple CSC types, we established a 3D-sphere-forming culture system of various CSC types, such as breast, colon and liver cancers (Figure 1A–F).

As mentioned above, drug resistance is one of the major characteristics of CSCs and a mechanism of tumor recurrence [28,29]. Therefore, selectively targeting and eliminating CSCs may be a more effective cancer therapeutic strategy to overcome recurrence [30]. Tumorigenic materials may stimulate the clonogenic abilities and stem cell-like properties of multiple CSC types. Importantly, CSCs can respond more robustly and sensitively to various tumorigenic stimuli than can non-stem cancer cells [31,32,33]. Consistent with this notion, the response to cytotoxic or antiproliferative drugs by CSCs matched the in vivo response better than did non-stem cancer cells, suggesting that the CSC-based screening platform may provide investigators with a more accurate prediction of in vivo responses to chemotherapeutic agents or carcinogens [34]. The different responses to tumorigenic chemicals or therapeutic drugs may result from the differential gene expression profiles of CSCs and non-stem cancer cells, as suggested by Hongisto et al. [35]. Our data revealed that the expression of an identified potential tumorigenic marker, SERPINB2, in multiple CSC types was significantly higher than that in non-stem cancer cells, although both non-stem cancer cells and CSCs exhibit basal expression of SERPINB2 (Figure 3D–F).

To identify a common marker for predicting potential tumorigenicity, we focused on a small group of genes that are functionally related to carcinogen-induced tumorigenic phenotypes in multiple types of human CSC models. One of these identified genes, SERPINB2, is significantly enhanced by TCDD exposure. Importantly, SERPINB2 expression has also been associated with tumor initiation, progression and metastasis in various cancers, such as bladder [11], colorectal [12], endometrial [13] and ovarian [14] cancers. These results suggest that SERPINB2 could serve as a sensitive marker for predicting tumorigenic responses to potential carcinogens. In the current study, we indeed observed increased expression of SERPINB2 after in vitro stimulation with TCDD in multiple types of CSCs (Figure 3G). Importantly, enhanced SERPINB2 levels were detected with most of the additional tested tumorigenic substances (Figure 4), suggesting that SERPINB2 can be used as a reliable universal marker for predicting tumorigenicity. Notably, the growth and stem cell-like properties of CSCs were successfully suppressed by SERPINB2 knockdown in multiple cancer types (Figure 7A–G). Consistent with our hypothesis, the results revealed that SERPINB2 positively regulates the clonogenic abilities and stem cell-like properties of multiple CSC types (Figure 7E–J). It is therefore quite possible that SERPINB2 mediates the effect of TCDD on various types of CSCs as a downstream regulator. Future studies investigating TCDD treatment of cells with or without SERPINB2 depletion would reinforce our hypothesis. Large-scale data analysis (Figure 5D–F) and our immunocytochemical analysis (Figure 6A–D) also revealed a strong relationship between the significantly increased expression of both SERPINB2 itself and constituents in its related signaling pathways and metastatic progression or recurrence in breast, colorectal and liver cancers. These results suggest that the stimulatory effects of tumorigenic materials on the self-renewal and stem cell-like properties of CSCs can be achieved by maintaining SERPINB2 expression. However, no individual cancer cell line is representative of specific cancer types and all functional studies in this study were performed with a single commonly used cell line for each cancer type.

## 4. Materials and Methods

### 4.1. Cancer Culture and Reagents

The human colon cancer cell line HT29, breast cancer cell line MDA-MB-231 and liver cancer cell line Huh7 were obtained from the Korean Cell Line Bank (Seoul, Korea) and were cultured in DMEM (Invitrogen, Grand Island, NY, USA) supplemented with 10% fetal bovine serum (FBS), 100 U/mL penicillin and 100 U/mL streptomycin (Lonza, Basel, Switzerland) at 37 °C in a 5% CO_2_ humidified incubator.

### 4.2. Flow Cytometry Analysis

To access the levels of Aldefluor, CD24, CD44 and CD133 positive populations, the cells were stained with Aldefluor kit (Stem Cell Technologies, Vancouver, Canada, Cat. 01700, dilution 1/40), PE conjugated CD24 (BD Bioscience, San. Jose, CA, USA, Cat. 555428, dilution 1/40), APC conjugated CD44 (BD Bioscience, Cat. 559942, dilution 1/40) and PE conjugated CD133 (MACS; Miltenyi Biotech, Sunnyvale, CA, USA, 130-080-081, dilution 1/40), respectively. Flow cytometry data were analyzed using FlowJo software (Tree Star, Ashland, OR, USA). The flow cytometry gates were established by staining with an isotype antibody or secondary antibody.

### 4.3. Tumorsphere Formation

HT29, MDA-MB-231 and Huh7 cells were resuspended in serum-free medium (Invitrogen) containing B27 (Invitrogen), 20 ng/mL EGF, 20 ng/mL bFGF (PeproTech) and 4 μg/mL heparin (Sigma-Aldrich, Saint Louis, MO, USA) and then plated onto plated onto six-well ultra-low attachment dishes (1 × 10^4^ cells/well) (Corning, NY, USA). The formed tumorspheres ≥ 100 μm from each replicate well (*n* ≥ 9 wells) were counted under an inverted microscope at 50× magnification using the Image-Pro program (Media Cybernetics, Rockville, MD USA). The percentage of cells with the ability to form spheres, termed the ‘tumorsphere formation efficiency (TSFE),’ was calculated as follows: ((number of spheres that formed/number of single cells that were plated) × 100).

### 4.4. Real-Time PCR

Total RNA was extracted using TRIzol reagent (Invitrogen) according to the manufacturer’s protocol. RNA purity was verified by measuring the 260/280 absorbance ratio. The first-strand cDNA was synthesized with 1 μg of total RNA using SuperScript II (Invitrogen) and one-tenth of the cDNA was used for each PCR mixture containing Express SYBR-Green qPCR Supermix (BioPrince, Seoul, Korea). Real-time PCR was performed using a Rotor-Gene Q (Qiagen, Hilden, Germany). The reaction was subjected to 40-cycle amplification at 95 °C for 20 s, 60 °C for 20 s and 72 °C for 25 s. The relative mRNA expression of the selected genes was normalized to that of PPIA and quantified using the ΔΔCT method. The sequences of the PCR primers are listed in Table 1.

### 4.5. Protein Isolation and Western Blot Analysis

The protein expression levels were determined by western blot analysis as previously described [36]. Cells were lysed in a buffer containing 50 mM Tris, 5 mM EDTA, 150 mM NaCl, 1 mM DTT, 0.01% NP 40 and 0.2 mM PMSF. The protein concentrations of the total cell lysates were measured by using bovine serum albumin as a standard. Samples containing equal amounts of protein were separated by sodium dodecyl sulfate-polyacrylamide gel electrophoresis (SDS-PAGE) and then transferred onto nitrocellulose membranes (Bio-Rad Laboratories, Hercules, CA, USA). The membranes were blocked with 5% skim milk in Tris-buffered saline containing Tween-20 at RT. Then, the membranes were incubated with primary anti-SERPINB2 (Abcam, MA, USA, ab47742) and β-actin (Abcam, MA, USA, ab189073) antibodies overnight at 4 °C and then with HRP-conjugated goat anti-rabbit IgG (BD Pharmingen, San Diego, CA, USA, 554021) and HRP goat anti-mouse IgG (BD Pharmingen, 554002) secondary antibodies for 60 min at RT. Antibody-bound proteins were detected using an ECL reagent.

### 4.6. SERPINB2 Knockdown

Small hairpin RNA (shRNA: accession No. NM_002575) targeting SERPINB2 and scrambled shRNA (shCon) were purchased from Bioneer (Daejeon, Korea). For efficient SERPINB2 transfection, reverse transfection was performed using Lipofectamine 2000 (Invitrogen) according to the manufacturer’s protocol. We chose the SERPINB2 shRNA that is most effective in mRNA levels from five shRNA designed from the target sequence and determined by qRT-PCR analysis. 

### 4.7. Ingenuity-Based SERPINB2-Related Genes Analysis

An “SERPINB2-related genes” analysis was performed with Ingenuity Pathway Analysis (IPA) version 2.0 software (Ingenuity Systems, Redwood City, CA, USA). Differentially expressed genes (*t*-test, *p* < 0.005) between metastatic cancer and non-metastatic cancer patients or relapse and relapse-free colorectal cancer patients were subjected to an “SERPINB2-related genes” analysis. The significance of each molecule was measured by Fisher’s exact test (*p* value), which was used to identify differentially expressed genes from the microarray data that overlapped with genes known to be regulated by a molecule. The activation score (z score) was used to show the status of predicted molecules by comparing the observed differential regulation of genes (“up” or “down”) in the microarray data relative to the literature-derived regulation direction, which can be either activating or inhibiting.

### 4.8. R2 Database Analysis

We used the R2: Genomics Analysis and Visualization Platform (http://hgserver1.amc.nl/cgi-bin/r2/main.cgi/) to analyze the expression levels of SERPINB2 between metastatic cancer and non-metastatic cancer patients or relapse and relapse-free breast (Booser cohort dataset), colorectal (Seiber cohort dataset) and liver (TCGA cohort dataset) cancer patients, respectively. The SERPINB2 value was log2 transformed and median centered. All of the graphics and statistic values were analyzed by GraphPad Prism 5.0 and *p*-values were calculated by a two-tailed Student’s t-test (*p* < 0.05). 

### 4.9. Gene set Enrichment Analysis (GSEA) of Metastatic or Recurrent Colon Cancer

Colorectal cancer clinical data were obtained and analyzed using the Seiber dataset (GSE25066 for breast cancer; GSE75316 for colorectal cancer; LIHC for liver cancer) [37] from ‘R2: Genomics Analysis and Visualization Platform (http://r2.amc.ml).’ The primary data are available from GEO (http://www.ncbi.nlm.nih.gov/geo/). A gene set enrichment analysis (GSEA) of the ranked gene list was performed using the Java implementation of GSEA obtained from http://www.broadinstitute.org/gsea/ (1000 permutations, minimum term size of 15 and maximum term size of 500). Differentially expressed genes between recurrent and non-recurrent or between metastatic and non-metastatic cancer were pre-ranked based on the mean fold change from the Seiber dataset (GSE25066 for breast cancer; GSE75316 for colorectal cancer; LIHC for liver cancer). The analysis included gene sets from MSigDB pathways, C2: all curated genes (c2.all.v5.0.symbols.gmt) or C6: oncogenic signature gene sets (c6.all.v5.0.symbols.gmt). The normalized enrichment score (NES) accounts for differences in gene set size. The FDR q-value (the probability that a gene set with a given NES represents a false-positive finding) was used to set a significance threshold.

### 4.10. Immunofluorescent Staining

Human tumor samples were obtained from colorectal cancer patients with informed consent and all of the experiments were approved by the Institutional Review Board of Gil Hospital (GCIRB-2013-66). Samples were fixed with 4% paraformaldehyde for fluorescent staining. Samples were permeabilized with 0.3 M glycine and 0.3% Triton X-100 and nonspecific binding was blocked with 2% normal swine serum (DAKO, Glostrup, Denmark). Staining was performed as described previously [38], using the primary SERPINB2 (Abcam, MA, USA, ab47742), CD44 (NOVUS, NBP1-48386), CD133 (NOVUS, NB120-16518) ALDH (Abcam, ab23375) antibodies. Alexa Fluor 488-conjugated rabbit IgG (Molecular Probes, Eugene, OR) was used to visualize SERPINB2. Samples were examined by fluorescence microscopy (Zeiss LSM 510 Meta, Oberkochen, Germany). The calculation of SERPINB2 expression was based on green fluorescence area and density divided by cell number, as determined from the number of DAPI-stained nuclei, in three randomly selected fields for each specimen from a total of three independent experiments. For quantitation, an arbitrary threshold was set to distinguish specific from background staining and this same threshold setting was applied to all the samples analyzed.

### 4.11. Statistical Analysis

The results are expressed as the mean ± the standard deviation (SD) of at least three independent experiments. The comparisons between the experimental groups and the corresponding controls were performed with GraphPad Prism 5.0 (GraphPad Software, San Diego, CA, USA) using one-way ANOVA. *p* < 0.05 was considered to indicate statistical significance.

## 5. Conclusions

Currently, the number of carcinogens is growing due to rapid industrialization, indicating the need for a better screening platform for predicting tumorigenicity. Our findings illuminate the critical requisites for the development of a target gene-based tumorigenicity screening platform, namely, the identification of target genes in response to various tumorigenic stimuli. In conclusion, based on the tumorigenic response gene SERPINB2, we can develop an in vitro screening platform. Significantly enhanced SERPINB2 expression levels were detected with some tested toxic substances and moderately increased levels were also detected with some other substances. However, several test substances barely increased SERPINB2 expression in human stem cells. These results warrant further prospective studies to verify the reliability of SERPINB2 as a universal marker for predicting tumorigenicity. Our CSC-based screening platform can provide valuable information on tumorigenic compounds that are not normally detected by other non-stem cancer cell-based systems. Furthermore, CSCs and target gene-based screening platforms might be a more effective strategy to improve screening rates of potential anticancer drugs with high efficacy. However, in vitro results sometimes do not properly reflect the actual in vivo conditions due to the lack of a tumor microenvironment and all functional experiments in this study were performed with in vitro platforms. Therefore, performing in vivo experiments with a mouse xenograft model in the future should be sufficient to overcome this major limitation.

## Figures and Tables

**Table 1:** Primer sequences for quantitative RT-PCR.

Gene	Gene Bank No.	Direction	Primer Sequence
Human NANOG	NM_024856	F	ACATGCAACCTGAAGACGTGTG
		R	CATGGAAACCAGAACACGTGG
Human OCT4	NM_002701	F	ACATCAAAGCTCTGCAGAAAGAACT
		R	CTGAATACCTTCCCAAATAGAACCC
Human SOX2	NM_003106	F	AAATGGGAGGGGTGCAAAAGAGGAG
		R	CAGCTGTCATTTGCTGTGGGTGATG
Human SERPINB2	NM_001143818	F	ACCCCCATGACTCCAGAGAACT
		R	GAGAGCGGAAGGATGAATGGAT
Human PPIA	NM_021130	F	TGCCATCGCCAAGGAGTAG
		R	TGCACAGACGGTCACTCAAA

## Supplementary Materials

The following are available online at https://www.mdpi.com/2072-6694/11/4/499/s1, Figure S1: Selection of a standard toxic compound for assessing stem cell toxicity, Figure S2: Identification of the concentration of TCDD to inhibit 50% of cell proliferation (IC50), Figure S3: The size of CSC spheres was significantly enlarged by TCDD treatment over a range of cell densities, Figure S4: Identification of the IC50 of multiple test substances on cell proliferation, Figure S5. The size of CSC spheres was significantly suppressed by SERPINB2 knockdown in all cell densities tested.
