# Supplementary material for: SERPINB2 Is a Novel Indicator of Cancer Stem Cell Tumorigenicity in Multiple Cancer Types
Source: Cancers (Basel). 2019 Apr 8;11(4):499. doi: 10.3390/cancers11040499 (PMC6520756; doi:10.3390/cancers11040499)
Supplement: Supplementary file 1 [file cancers-11-00499-s001.pdf]

Supplementary Materials: SERPINB2 is a Novel Indicator of Cancer Stem Cell Tumorigenicity in Multiple Cancer Types

Na-Hee Lee, Se-Ra Park, Jin Woo Lee, Soyi Lim, Seung-Ho Lee, Seungyoon Nam, Dong Young Kim, Seung Yeon Ha, In-Sun Hong and Hwa-Yong Lee

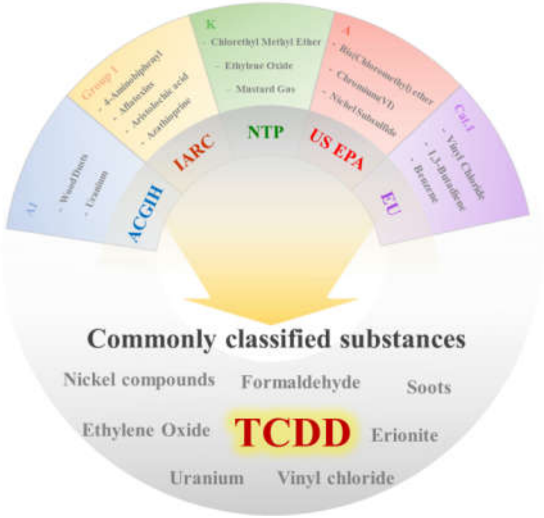

Figure S1. Selection of a standard toxic compound for assessing stem cell toxicity. A standard test compound was chosen from the list of top-ranked compounds according to the common hazardous material classification of five authorized international organizations: the IARC (International Agency for Research on Cancer), ACGIH (Association Advancing Occupational and Environmental Health), NTP (National Toxicology Program), US EPA (Environmental Protection Agency), and EU (European Union). Of the top-ranked hazardous materials, TCDD was selected as a standard test compound due to its severe toxicity and tumorigenicity.

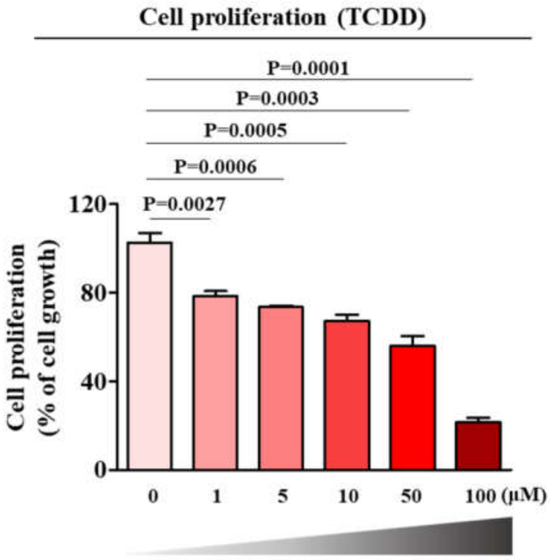

Figure S2. Identification of the concentration of TCDD to inhibit 50% of cell proliferation (IC_{50}). The approximate IC_{50} value of TCDD was determined by testing the responses of normal human cells to multiple concentrations from 1 μM to 100 μM for 48 hours using an MTT assay. The cell viability (%) was calculated as the percent of the vehicle control.

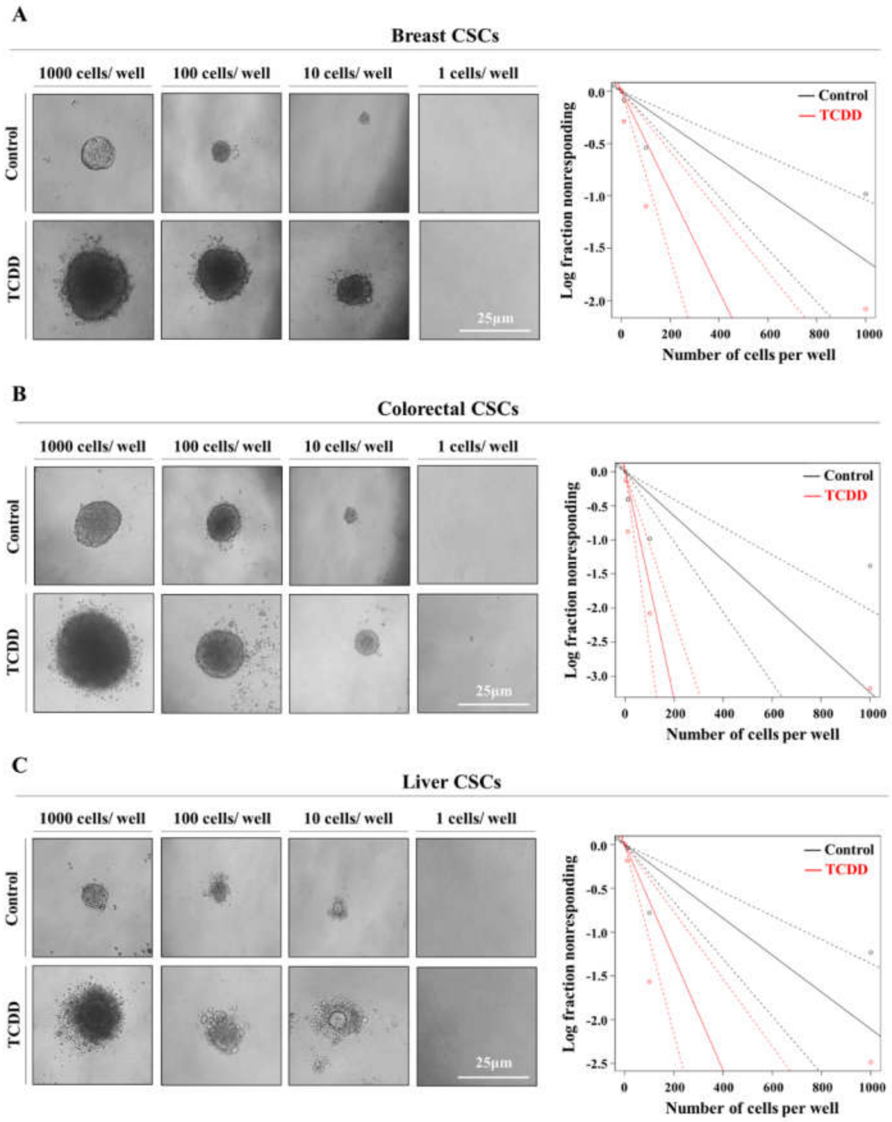

Figure S3. The size of CSC spheres was significantly enlarged by TCDD treatment over a range of cell densities. Human breast (MDA-MB-231), colorectal (HT29), and liver (Huh7) cancer cells treated with TCDD (10 nM) were seeded at 1, 10, 100, and 1000 cells/well. After 21 days of culture, the number of wells that were positive or negative for the presence of growing tumor spheres was determined. The results represent the mean ± SD from three independent experiments.

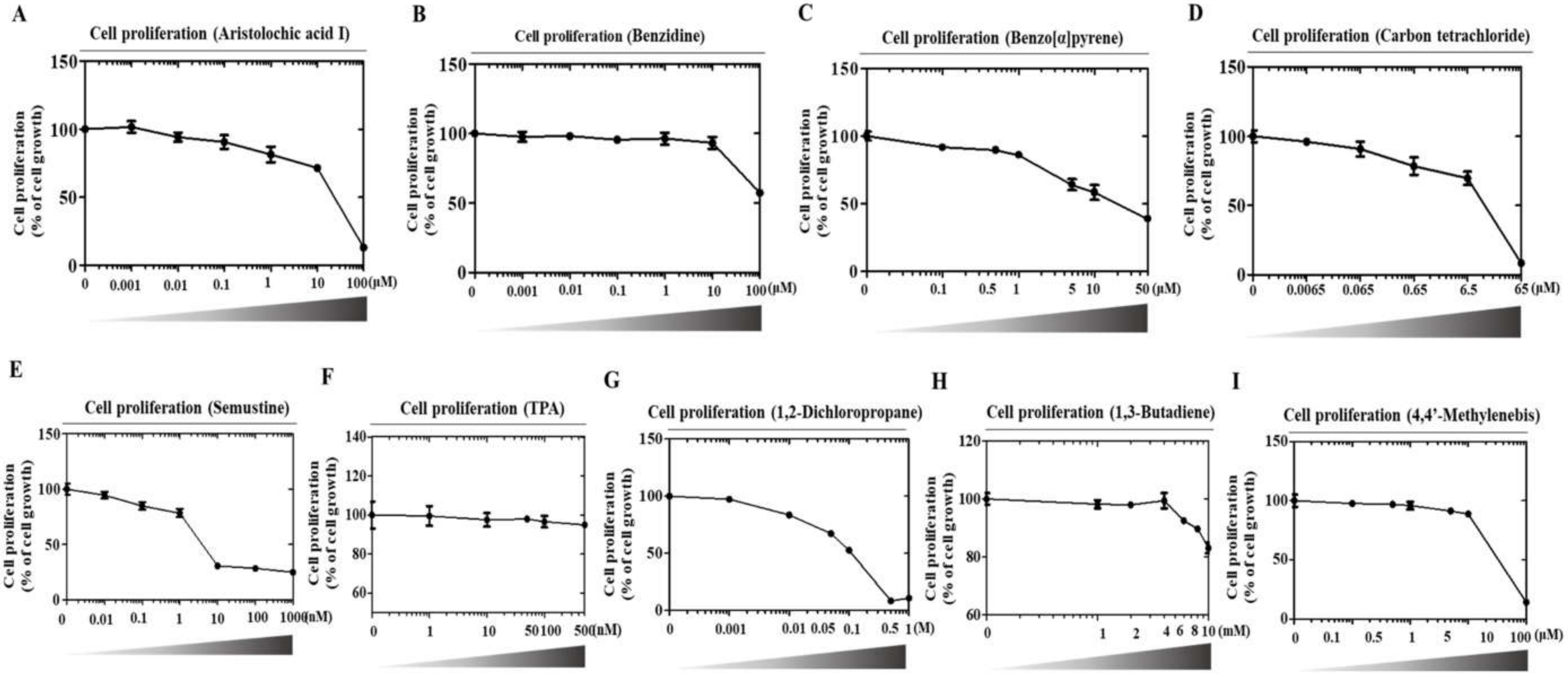

Figure S4. Identification of the IC_{50} of multiple test substances on cell proliferation. The inhibition of cell viability using multiple test substances, including aristolochic acid I, benzidine, benzo[a]pyrene, carbon tetrachloride, semustine, TPA, 1,2-dichloropropane, 1,3-butadiene, and 4,4-methylenebis, for 48 hours was determined using an MTT assay in normal human cells **(A–I)**. The cell viability (%) was calculated as the percent of the vehicle control.

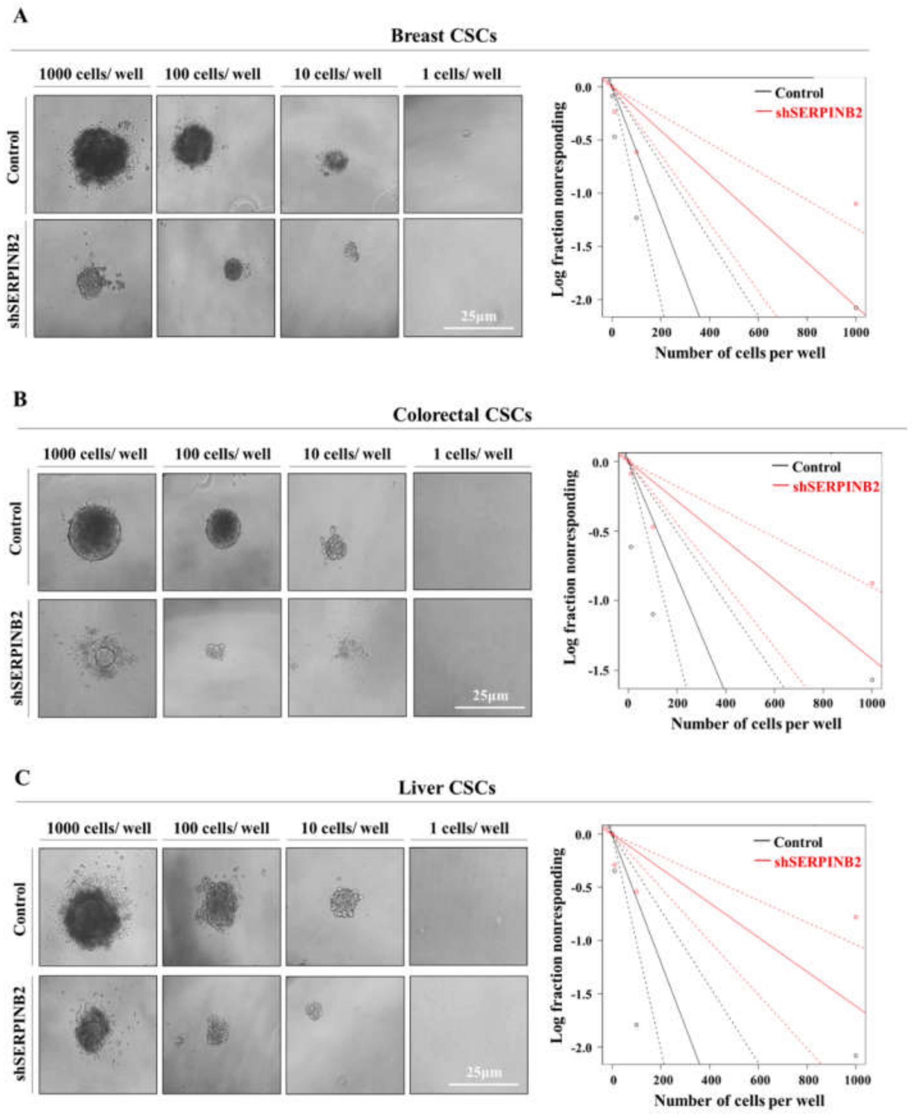

Figure S5. The size of CSC spheres was significantly suppressed by SERPINB2 knockdown in all cell densities tested. Human breast (MDA-MB-231), colorectal (HT29), and liver (Huh7) cancer cells transfected with shRNA targeting SERPINB2 were seeded at 1, 10, 100, and 1000 cells/well. After 21 days of culture, the number of wells that were positive or negative for the presence of growing tumor spheres was determined. The results represent the mean ± SD from three independent experiments.

© 2019 by the authors. Licensee MDPI, Basel, Switzerland. This article is an open access article distributed under the terms and conditions of the Creative Commons Attribution (CC BY) license (http://creativecommons.org/licenses/by/4.0/).
